# Colonic polyp histopathology and location in a community-based sample of older adults

**DOI:** 10.1186/s12876-016-0497-1

**Published:** 2016-08-02

**Authors:** Heather S. Laird-Fick, Gurveen Chahal, Ade Olomu, Joseph Gardiner, James Richard, Nikolay Dimitrov

**Affiliations:** 1 Department of Medicine, Michigan State University College of Human Medicine, East Lansing, MI 48824 USA; 2 Department of Epidemiology and Biostatistics, Michigan State University College of Human Medicine, East Lansing, MI 48824 USA; 3 Department of Pathology, EW Sparrow Hospital, CAP-Labs, Lansing, MI 48912 USA

**Keywords:** Colon polyps, Sessile serrated adenomas, Colonoscopy, Epidemiology

## Abstract

**Background:**

Colorectal cancer and its precursors are highly prevalent in developed countries. Estimates in the available literature for prevalence of right-sided-only lesions vary between 20.5 and 48.1 %, with association with female gender and advancing age. Since the original polyp studies, premalignant potential of sessile serrated adenomas has been described and screening utilization of colonoscopy in men, women, and older adults has increased. This study describes the histopathology and distribution of colorectal polyps by age and gender in the post-screening era.

**Methods:**

A registry of biopsies performed during colonoscopy for adults aged 50+ years in 2002-2012 was created using pathology reports from an independent, regional laboratory. Age, histopathology, and polyp location(s) were included. A subgroup analysis was performed for sessile serrated adenomas for 2007-2012. Distributions of histopathology and polyp location were described by age and gender. Statistical comparisons are made using chi-square tests.

**Results:**

13,881 patients (55.5 % male, 44.5 % female), aged 50-95 years (median = 62) were identified. Most patients (59.9 %) had adenomas. Single and multiple adenomas were more common in men than women (57.7 % vs 42.3 %, *p* < .0001 and 62.2 % vs 37.8 %, *p* < .001), and with advancing age (60.4 % for ages 50- < 60, 63.4 % for ages 60- < 70, 65 % for ages 70- < 80, and 68.9 % for ages >80). Villous adenomas (n = 545; 3.6 %), dysplasia (*n* = 49; 0.4 %), and invasive carcinoma (*n* = 22; 0.2 %) were rare. Sessile serrated adenomas were uncommon (*n* = 417, 4.5 %), with greater prevalence in women than men (5.1 % vs 4 %, *p* = 0.02). Patients aged 70- < 80 were more likely to have multiple polyps than those aged 50- < 60 (OR 1.17, 95 % CI 1.03–1.32, *p* = 0.018 and OR 1.27, 95 % CI 1.10–1.46, *p* = .001). Most polyps were from ascending and/or transverse colon (*n* = 8095; 58.3 %). When location was stratified by sex only, men had more polyps than women at each location except the sigmoid and rectum. Further stratification by age of location and sex revealed statistically significant differences (age 50- < 60, *p* < .0001, age 60- < 70, *p* = .0227, age 70- < 80, *p* = .0298, age 80+, *p* = .0018).

**Conclusions:**

This large community-based sample contributes to understanding of colonic neoplasia. The high prevalence of right and transverse lesions supports ongoing use of colonoscopy over sigmoidoscopy for screening examinations.

## Background

Colorectal cancer is the second leading cause of cancer deaths in the United States (US) [1]. Screening for premalignant colorectal lesions and asymptomatic disease can decrease disease-associated morbidity and mortality. The United States Preventive Services Task Force recommends screening average risk adults aged 50 to 75 years for colorectal cancer using one of several modalities – fecal occult blood, sigmoidoscopy, or colonoscopy [2]. The same tests are recommended for screening higher risk patients, such as those with family history of colorectal cancer, although testing may be offered at younger ages. Continued inclusion of sigmoidoscopy, while controversial among clinicians, is predicated upon a low rate of lesions isolated to the ascending or transverse colon, lower cost, and lower complication rates.

The National Polyp Study, conducted between 1980 and 1990, is the landmark trial which underlies current screening guidelines. Unfortunately, the study’s results may not represent the epidemiology of colonoscopy findings in today’s general population. Demographics differed; few adults older than age 75 years were included, fewer than half of participants were women, and ethnic minorities were underrepresented [3]. Furthermore, the malignant potential of the sessile serrated polyp was not described until 2007 [4], and therefore some patients at risk for subsequent cancer were likely missed.

In studies from the US and abroad, the distribution of polyps and carcinoma on colonoscopies has varied, with 48.5-60 % occurring in the left colon and rectum, 20.5–48.1 % on the right and transverse colon, and 24.5–28.7 % with synchronous lesions in both the right and left sided colon [5–8]. Female gender and advancing age have been associated with greater likelihood of right-sided lesions [5]. The reason for the variability of findings is unclear, but might be due to differences in population risks for different countries (e.g. Austria, Saudi Arabia, and the US), inclusion of screening versus diagnostic colonoscopies, or changes in presentation due to ongoing screening efforts.

This study describes the histopathology and distribution of colorectal polyps by age and gender in the post-screening era using a local registry of colonoscopy biopsy samples obtained between 2002 and 2012. A subgroup analysis of sessile serrated adenomas for the years 2007 to 2012 is also reported.

## Methods

A registry of colonoscopy pathology reports for adults, aged 50 to 100 years, obtained between the years 2002 to 2012 was created. Reports were drawn from an accredited, CLIA-certified, independent laboratory specializing in surgical pathology and serving community-based hospitals and surgical centers in the mid-Michigan region. The laboratory employed strict quality controls, including: 1) active review and application of updated histopathological criteria (including that for sessile serrated adenoma), 2) second pathologist confirmation of all reports of carcinoma or advanced neoplasia, and 3) second pathologist review if any uncertainty of the pathologic diagnosis existed.

Cases were defined as all biopsy samples submitted for processing on a single patient for a single date. The registry included the following information: case identifier, date of the procedure, patient date of birth, patient gender, the site within the colon from which the biopsy specimen was taken, and the free text of the histopathology report. Because the reports came from an independent laboratory, additional patient-level data was not available, including demographic information (race/ethnicity, socioeconomic status), and family or personal history of colon cancer or colorectal polyps. Cases were not differentiated based upon indication for the procedure (e.g. screening versus diagnostic testing).

Case data were coded for age at time of procedure (in years), most advanced level of histopathology identified, and region(s) of the colon sampled.

Sessile serrated adenomas (SSAs) were not reported as a distinct entity for the first few years of our registry, but by 2007 the laboratory had firmly established internal criteria and protocols for diagnosing them. Therefore a separate analysis was done for the years 2007 to 2012. Cases found to have both sessile serrated adenomas and other histological changes (adenoma, villous adenoma, dysplasia, or invasive carcinoma) were included, although they had been coded under the other histology in the primary analysis.

Demographic statistics on colon biopsies by age and gender were summarized using frequencies and percentages. The distribution of histopathology, polyp morphology and polyp location were compared by age and gender by chi-square tests. With multiple versus single histology as outcome, logistic regression was used to assess the association of age and gender, with results summarized by odds ratios and associated 95 % confidence intervals.

The de-identified clinical dataset supporting the conclusions of this article is available upon request to the corresponding author.

The study was approved as exempt by the Michigan State University institutional review board (IRB # x12-1297e).

## Results

A total of 13,881 patients had one or more biopsy specimens taken during colonoscopies that showed polyp(s) or invasive carcinoma. A total of 7,705 (55.5 %) were male and 6,176 (44.5 %) female. Ages ranged from 50 to 95 years, with a mean (SD) of 62.8 (9.2) years and median of 62 years. Females were slightly older (mean age 63.3 years) than males (mean age 62.5 years) and due to the large sample size, the difference was significant (*p* < .0001). Differences in gender were seen in each age category (Table 1).

**Table 1:** Distribution of Sex by Age among 13,881 patients

Age	Total^b^, n (%)	Sex^a^		p-value
		Female, n (%)	Male, n (%)	
50- < 60	5828 (42.0)	2509 (43.1)	3319 (57.0)	<.0001
60- < 70	4636 (33.4)	2048 (44.2)	2588 (55.8)	<.0001
70- < 80	2751 (19.8)	1251 (45.5)	1500 (54.5)	<.0001
80-95	666 (4.8)	368 (55.3)	298 (44.7)	0.007
Overall	13881 (100.0)	6176 (44.5)	7705 (55.5)	<.0001
Mean (SD), years	62.8 (9.2)	63.3 (9.5)	62.5 (8.9)	<.0001

^a^Percent in row might not sum to 100 due to rounding. Chi-square tests compare

sex distribution by age category, and overall. Mean ages compared by Wilcoxon test

^b^Percent of all patients (*n* = 13,881)

### Histopathology

Adenomatous polyps, whether singular or multiple, were the most common pathological finding (*n* = 8,305; 59.9 %), followed by hyperplastic polyps (*n* = 4,600; 33.1 %). In contrast, villous adenomas (n = 545; 3.6 %), moderate to high grade dysplasia (*n* = 49; 0.4 %) and invasive carcinoma (*n* = 22; 0.2 %) were rare (Table 2).

**Table 2:** Distribution of Sex by Histology among 13,881 patients

Histopathology	Total^b^ n (%)	Sex^a^		p-value
		Female n (%)	Male n (%)	
Hyperplastic, single	3296 (23.7)	1699 (51.6)	1597 (48.4)	0.077
Adenoma, single	5449 (39.3)	2307 (42.3)	3142 (57.7)	<.0001
Villous adenoma, single	175 (1.3)	73 (41.7)	102 (58.3)	0.028
Sessile serrated adenoma, single	215 (1.6)	109 (50.7)	106 (49.3)	0.838
Dysplasia, single	23 (0.2)	6 (26.1)	17 (73.9)	0.022
Invasive carcinoma, single	10 (0.1)	4 (40.0)	6 (60.0)	0.527
Hyperplastic, multiple	1304 (9.4)	662 (50.8)	642 (49.2)	0.580
Adenoma, multiple	2856 (20.6)	1078 (37.8)	1778 (62.2)	<.0001
Villous adenoma, multiple	370 (2.3)	149 (40.3)	221 (59.7)	0.0002
Sessile serrated adenoma, multiple	145 (1.0)	78 (53.8)	67 (46.2)	0.361
Dysplasia, multiple	26 (0.2)	5 (19.2)	21 (80.8)	0.002
Invasive carcinoma, multiple	12 (0.1)	6 (50.0)	6 (50.0)	1.00
Overall single	9168 (66.1)	4198 (45.8)	4970 (54.2)	<.0001
Overall multiple	4713 (34.0)	1978 (42.0)	2735 (58.0)	<.0001
Total	13881 (100.0)	6176 (44.5)	7705 (55.5)	<.0001

^a^Percent in row might not sum to 100 due to rounding. ^b^Percent of total patients

**Fig. 1:**
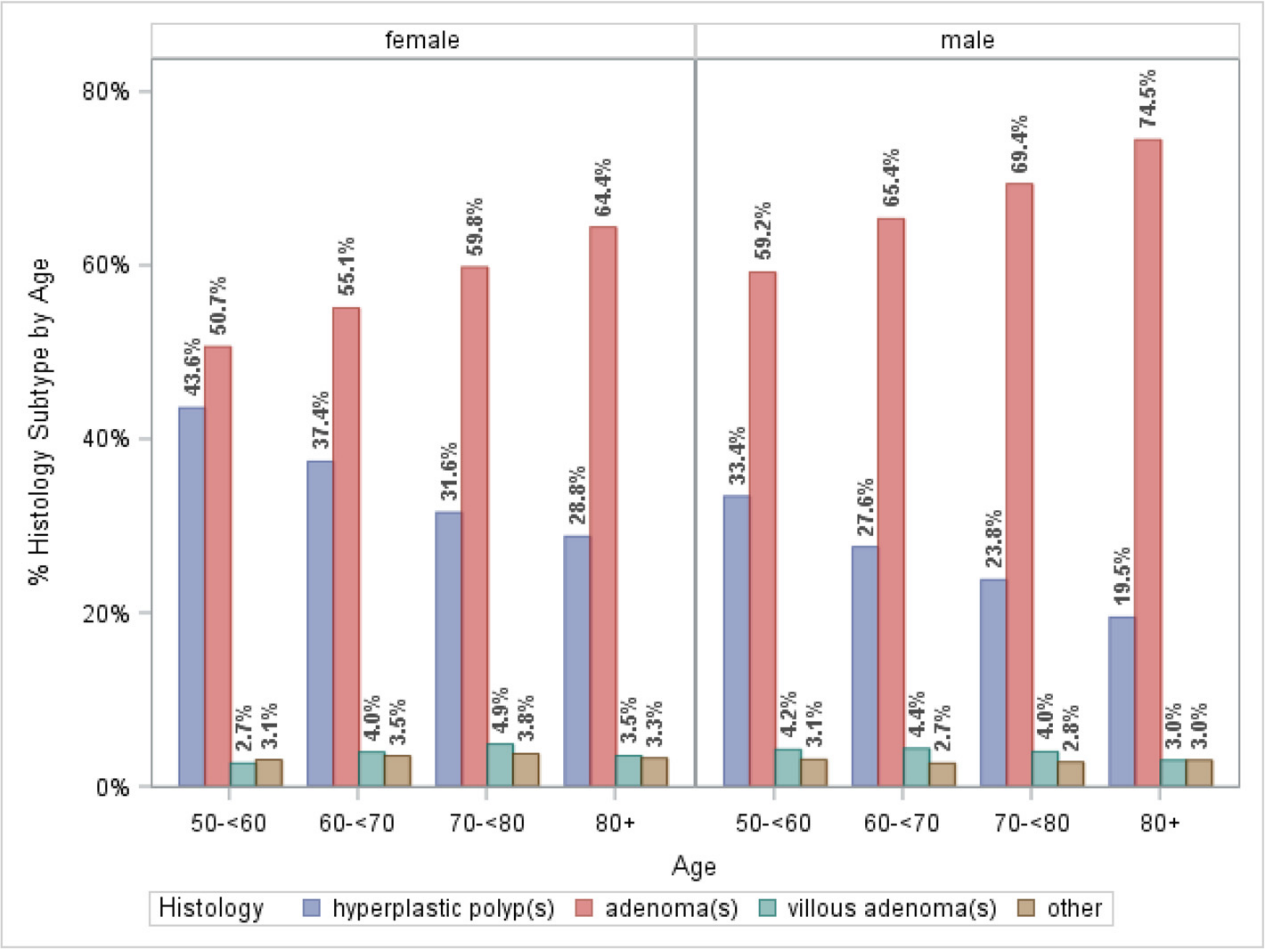
Distribution of histology by age stratified by sex. Comparisons of the distribution of polyp histology by sex in each age group were significant: Age 50- < 60 years, *p* < .0001; age 60- < 70 years, *p* < .0001; age 70- < 80, *p* < .0001; age 80+ years, *p* = .0379

Typical adenomas, whether single or multiple, were more common among males (*n* = 4920; 63.9 %) than females (*n* = 3385; 54.9 %) (*p* < .0001 for comparison). Single hyperplastic polyps more common among women (*n* = 1699; 51.6 %) than men (*n* = 1597; 48.4 %) (*p* = 0.077); there was no significant difference by sex for multiple hyperplastic polyps (*p* = 0.580) (Fig. 1, Table 2).

**Fig. 2:**
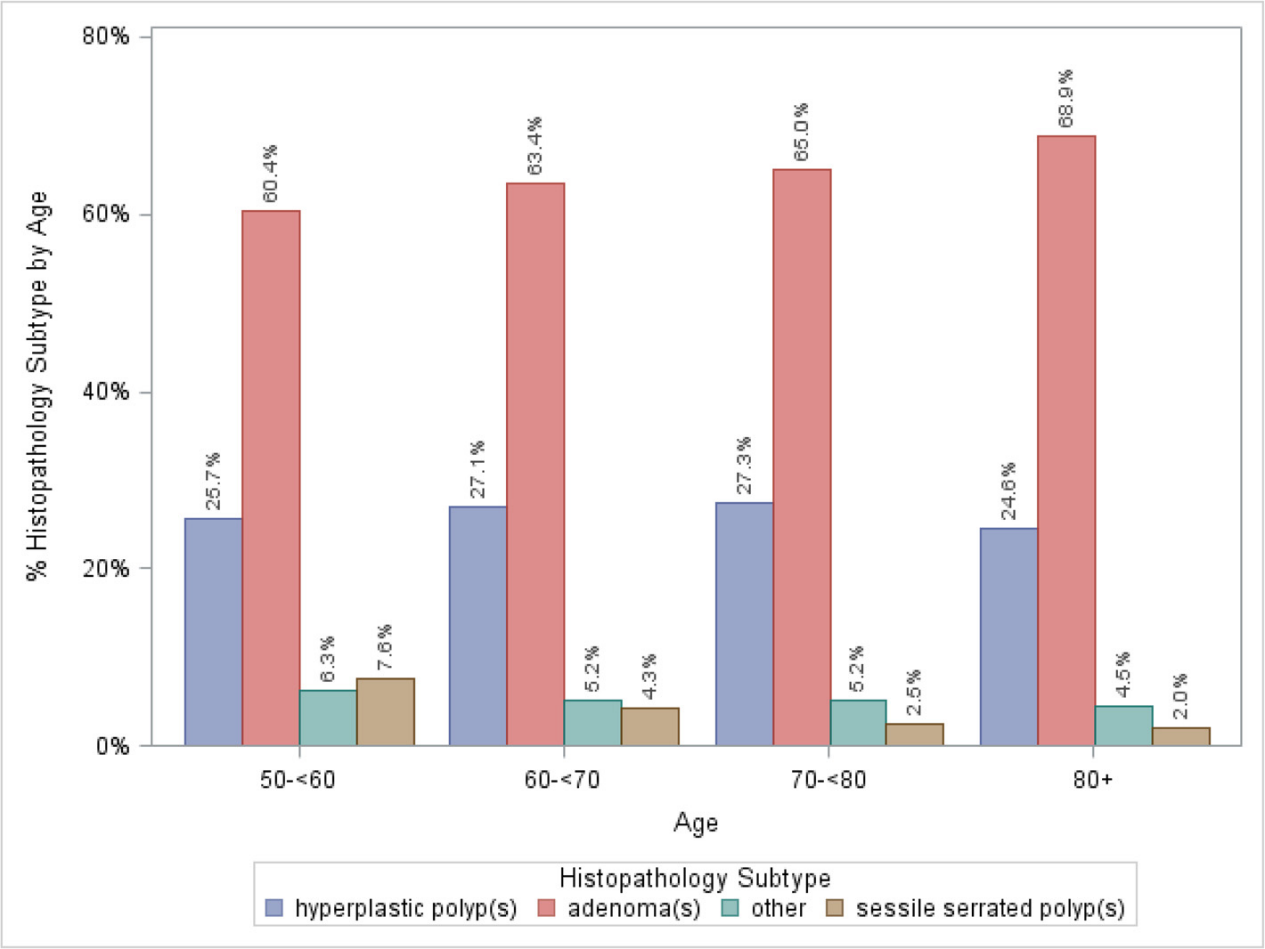
Distribution of histology by age for subgroup analysis, 2007-2012. *p* < .0001 for distribution of histology across age groups

Trends in histopathology based on age were seen as well. Adenomas were the most common finding across all age groups, and prevalence increased with increasing age (Fig. 2).

Between 2007 and 2012, SSAs were found in a total of 417 patients (4.5 %), (Table 3). There was no statistical difference by sex, in overall SSA or within SSA types. However, amongst all 9301 patients, prevalence of SSA was higher in females (*n* = 215; 5.1 %) than in males (*n* = 202; 4.0 %) (*p* = 0.02). Prevalence of SSA was also greater in patients ages 50- < 60 years than older adults (7.6 % versus 2.0-4.3 %. *p* < .0001 for distribution of histology by age) as seen in Fig. 2.

**Table 3:** Prevalence of sessile serrated adenomas, 2007-2012 in 9301 patients

Histology	Total^a^ n (%)	Sex		p-value
		Female n (%)	Male n (%)	
Solitary	215 (2.3)	109 (50.7)	106 (49.3)	0.838
Multiple	145 (1.6)	78 (53.8)	67 (46.2)	0.361
SSA Other	57 (0.6)	28 (49.1)	29 (50.9)	0.895
Overall	417 (4.5)	215 (51.6)	202 (48.4)	0.524
Non SSA	8884 (95.5)	4016(45.2)	4868 (54.8)	<.0001

^a^Percent of patients (*n* = 9301)

### Number of polyps

Polyps were classified as single or multiple in the registry. The prevalence of multiple polyps was higher in men (*n* = 2,735; 35.5 %) than in women (*n* = 1,978; 32.0 %), *p* < .0001. Multiple polyps were also more common with increasing age: age 50–59 years, 32.3 %; 60–69 years, 34.3 %; 70–79 years, 36.5 %; and 80+ years, 35.7 % (*p* = .001). Using age 50–59 years and sex as referent categories, the odds of having multiple (vs single) polyps was higher at older age, while female sex had lower odds relative to males (Table 4). Both age and sex effects were significant; an age by sex interaction was not found.

**Table 4:** Odds ratios and 95 % confidence intervals for association of age with multiple versus single polyp classification in 13,881 patients

	Histology Frequency	Odds Ratio	95 % Confidence Limits		p-value
	Multiple/Single				
Females: Age					
50- < 60 referent	755/1754	1.00			
60- < 70	659/1389	1.10	0.97	1.25	0.130
70- < 80	441/810	1.27	1.10	1.46	0.001
80-95	123/245	1.17	0.92	1.47	0.195
Males: Age					
50- < 60 referent	1127/2192	1.00			
60- < 70	931/1657	1.09	0.98	1.22	0.106
70- < 80	562/938	1.17	1.03	1.32	0.018
80-95	115/183	1.22	0.96	1.56	0.107
Adjusted^a^					
Male referent	2735/4970	1.00			
Female	1978/4198	0.85	0.79	0.91	<.0001
Age, 5 yr increase	…	1.05	1.03	1.07	<.0001

^a^Estimates from multiple logistic regression with Sex and Age

### Location

**Fig. 3:**
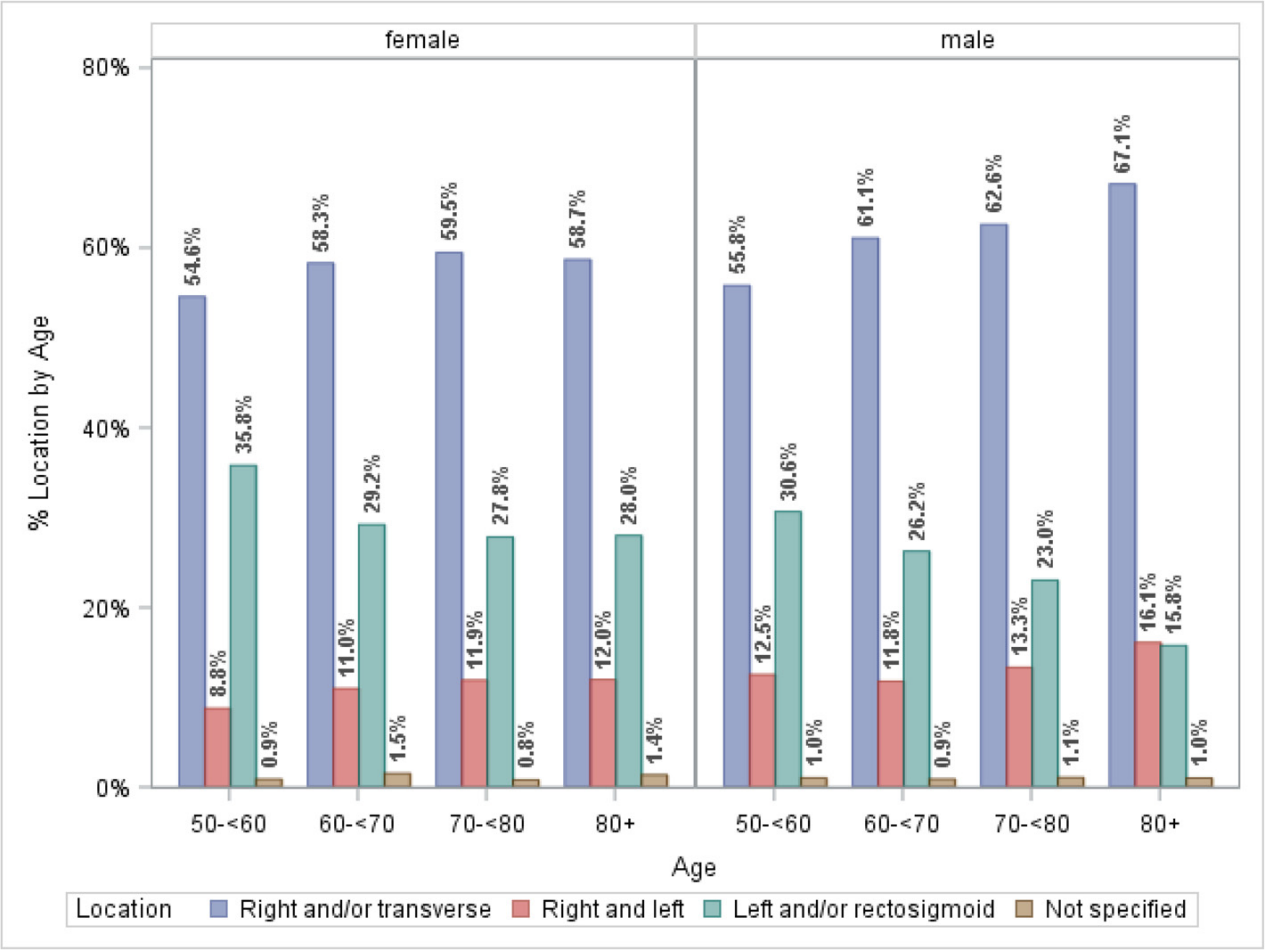
Distribution of polyp location by age stratified by sex. In each age group, there are statistically significant differences of the distribution of location by sex. Age 50- < 60 years, *p* < .0001; age 60- < 70 years, *p* = .0227; age 70- < 80 years, *p* = .0298; age 80+ years, *p* = .0018

More than half the patients had biopsy specimens isolated within either the right colon (*n* = 3,943; 28.4 %), the transverse colon (*n* = 3,288; 23.7 %), or a combination of the two (*n* = 864; 6.2 %) (Table 5) – regions beyond the reach of a sigmoidoscope. Only 29.1 % of patients had specimens isolated to the left side of the colon: left colon (*n* = 1,379; 9.9 %), sigmoid colon (*n* = 2,382; 17.2 %), rectum (*n* = 94; 0.7 %), or a combination of these sites (*n* = 180; 1.3 %). A minority of patients (*n* = 1,607; 11.6 %) had polyps on both the left side of the colon and either the ascending or transverse colon. Information about the site of the biopsy specimen was missing for 144 patients (1.0 %). There was a small but statistically significant difference in distribution by gender and age (Fig. 3).

**Table 5:** Distribution of polyps by location among 13,881 patients

Location	Distance from rectum (in centimeters)	Total^b^ n (%)	Sex^a^		p-value
			Female n (%)	Male n (%)	
Right	132–147	3943 (28.4)	1842 (46.7)	2101 (53.3)	<.0001
Transverse and flexures	82–132	3288 (23.7)	1351 (41.1)	1937 (58.9)	<.0001
Ascending and transverse	n/a	864 (6.2)	330 (38.2)	534 (61.8)	<.0001
Left	57–82	1379 (9.9)	645 (46.8)	734 (53.2)	0.017
Sigmoid	15–57	2382 (17.2)	1178 (49.5)	1204 (50.6)	0.594
Rectum	4 to 14	94 (0.70)	48 (51.1)	46 (48.9)	0.837
Left and (transverse or ascending)	n/a	1607 (11.6)	638 (39.7)	969 (60.3)	<.0001
Left and sigmoid	n/a	180 (1.3)	76 (42.2)	104 (57.8)	0.037
Not specified	n/a	144 (1.0)	68 (47.2)	76 (52.8)	0.510
Total		13881 (100.0)	6176 (44.5)	7705 (55.5)	<.0001

^a^Percent in row might not sum to 100 due to rounding. ^b^Percent of total patients

## Discussion

In this community based sample of adults aged 50 and older, adenomas were the most common histopathological finding (*n* = 8,305; 59.9 %), with higher prevalence in males (*p* < .0001) and increasing prevalence by age in either sex (*p* < .0001).

Isolated hyperplastic polyps were the second most common finding (*n* = 4,600; 33.1 %). The presence of multiple polyps was positively associated with male sex (*p* < .0001) and increasing age (*p* = .001). 58.3 % of patients had no lesions within reach of a typical sigmoidoscope; while there were statistically significant differences in polyp location based on age and gender, the absolute differences were small and unlikely to be of clinical significance.

These results differ from previous reports in several important ways. Most studies have reported a preponderance of left-sided polyps [5, 6, 8], with right-sided ones more common in women [5]. In contrast, most patients in this study had right-sided polyps, regardless of gender and age. In addition, few patients (*n* = 22; 0.2 %) in this registry had invasive carcinoma, validating results from Odom et al who reported a 0.3 % rate of invasive carcinoma in single community hospital between 1999 and 2002 [9]. Other studies have found higher rates, ranging from 1.1 % in a prospective four year screening study in Austria [6], to 6 % in the enrollment phase of the National Polyp Study [3]. In contrast to Odom et al, this study found more villous adenomas (3.6 %, versus 1.0 %) [9], and provides data on sessile serrated adenomas.

Information about the epidemiology of SSAs is limited. Prevalence has been reported between 0.6–13.8 % of resected polyps [7, 10, 11]. Coexisting adenomatous polyps were found in nearly half of patients in one series [11]. The present study found an intermediate prevalence (4.5 %); 86.3 % of these patients did not have other adenomatous polyp types present. Some studies [7, 10], but not all [11], have reported a female preponderance. This study found no statistical difference by sex, in overall SSA or within SSA types. However, amongst all 9301 patients, prevalence of SSA was higher in females (*n* = 215; 5.1 %) than in males (*n* = 202; 4.0 %) (*p* = 0.02).

This study has numerous strengths. It utilized a large, community based sample with inclusion of significant numbers of women and older adults. Experts in surgical pathology interpreted all specimens, and their reports provided greater detail than available through most epidemiologic studies. Limitations include lack of information about the race/ethnicity of patients, or the indications for individual studies (i.e. diagnosis in symptomatic patients, screening for average or high risk, or surveillance with personal history of polyposis). Some patients may have also been included more than once, if they underwent repeat colonoscopies in the ten year period.

## Conclusions

Right-sided and transverse polyps were more common in this community-based registry than previously reported, supporting whole-colon screening (e.g. colonoscopy) as opposed to regional evaluation (e.g. sigmoidoscopy). Invasive carcinoma was rare, but villous adenomas more common than in another community-based study [9]. Sessile serrated adenomas were uncommon (*n* = 417, 4.5 %), with greater prevalence in women than men (5.1 % vs 4 %, *p* = 0.02) and adults aged 50- < 60 years (Fig. 2), warrant endoscopist vigilance for flat lesions in these populations.

## Abbreviations

SD, Standard Deviation; SSA, Sessile serrated adenoman; US, United States
